# Statistical assessment of reliability of anthropometric measurements in the multi-site South African National Dietary Intake Survey 2022

**DOI:** 10.1038/s41430-024-01449-1

**Published:** 2024-05-14

**Authors:** Sanja Nel, Jeroen de Man, Louise van den Berg, Friedeburg Anna Maria Wenhold

**Affiliations:** 1 University of Pretoria, Department of Human Nutrition, Faculty of Health Sciences, Pretoria, South Africa; 2 University of Pretoria Research Centre for Maternal, Fetal, Newborn & Child Health Care Strategies, Kalafong Hospital, Atteridgeville, South Africa; 3 South African Medical Research Council (SA MRC) Maternal and Infant Health Care Strategies Unit, Kalafong Hospital, Atteridgeville, South Africa; 4 School of Public Health, Faculty of Community and Health Sciences, University of the Western Cape, Cape Town, South Africa; 5 Department of Family Medicine and Population Health, University of Antwerp, Antwerp, Belgium; 6 University of the Free State, Department of Nutrition and Dietetics, School of Health and Rehabilitation Sciences, Faculty of Health Sciences, Bloemfontein, South Africa

**Keywords:** Epidemiology, Nutrition

## Abstract

**Background:**

Anthropometric data quality in large multicentre nutrition surveys is seldom adequately assessed. In preparation for the South African National Dietary Intake Survey (NDIS-2022), this study assessed site leads’ and fieldworkers’ intra- and inter-rater reliability for measuring weight, length/height, mid-upper arm circumference (MUAC), waist circumference (WC) and calf circumference (CC).

**Methods:**

Standardised training materials and measurement protocols were developed, and new anthropometric equipment was procured. Following two training rounds (12 site lead teams, 46 fieldworker teams), measurement reliability was assessed for both groups, using repeated measurements of volunteers similar to the survey target population. Reliability was statistically assessed using the technical error of measurement (TEM), relative TEM (%TEM), intra-class correlation coefficient (ICC) and coefficient of reliability (R). Agreement was visualised with Bland-Altman analysis.

**Results:**

By %TEM, the best reliability was achieved for weight (%TEM = 0.260–0.923) and length/height (%TEM = 0.434–0.855), and the poorest for MUAC by fieldworkers (%TEM = 2.592–3.199) and WC (%TEM = 2.353–2.945). Whole-sample ICC and R were excellent ( > 0.90) for all parameters except site leads’ CC inter-rater reliability (ICC = 0.896, *R* = 0.889) and fieldworkers’ inter-rater reliability for MUAC in children under two (ICC = 0.851, R = 0.881). Bland-Altman analysis revealed no significant bias except in fieldworkers’ intra-rater reliability of length/height measurement in adolescents/adults ( + 0.220 (0.042, 0.400) cm). Reliability was higher for site leads vs. fieldworkers, for intra-rater vs. inter-rater assessment, and for weight and length/height vs. circumference measurements.

**Conclusion:**

NDIS-2022 site leads and fieldworkers displayed acceptable reliability in performing anthropometric measurements, highlighting the importance of intensive training and standardised measurement protocols. Ongoing reliability assessment during data collection is recommended.

## Introduction

Anthropometric measurements are generally considered easy to perform, but in reality, accurate and reliable measurement is challenging. Anthropometric data quality in multi-site national Demographic and Health Surveys varies greatly within and between countries [1]. Substandard anthropometric data quality compromises the accuracy of the results (including estimates of population nutrition status), while potentially attenuating or exaggerating associations between anthropometric data and other variables of interest. Furthermore, questionable data quality limits the comparability of data over time, and within and between studies. A recent synthesis of scoping reviews conducted in low- and middle-income countries found wide between-study variation in malnutrition prevalence estimates in school-age children: in South African studies, reported prevalences ranged from 10–37% for stunting, 18–34% for wasting, and 4–81% for thinness [2]. While temporal and sociodemographic differences account for some variation, the possible contribution of inconsistent or inappropriate measurement techniques cannot be ignored.

Reliability (also called repeatability or reproducibility) describes the degree to which repeated measurements of the same (unchanged) parameter yield the same results. Reliability may be assessed for repeated measurements by one measurer (intra-rater reliability) or for measurements of the same parameter by two or more measurers (inter-rater reliability). The degree of variability in repeated measurements is inversely related to reliability, providing the basis for quantitative assessment [3].

Many factors affect the reliability of anthropometric measurements. Equipment-related error can be minimised by using identical high-quality equipment across all sites, preferably newly purchased, and performing daily verification of accuracy and periodic calibration. Consistent, appropriate measurement technique is essential. Measurement reliability can be affected by several aspects of technique, including measurement site (e.g., for waist circumference (WC) [4–6]), equipment choice (e.g. types of tape measures) and the exact techniques used (e.g. infant length). In a multi-site study, these factors should be standardised a priori when developing measurement protocols, and fieldworkers trained accordingly [7–10]. Standardised, uniform fieldworker training should be followed by practical assessment of measurement techniques and statistical assessment of reliability. Apart from fieldworker competence, the ease of taking precise measurements depends on participant factors (e.g. participant cooperation), age and body size [11] and the anthropometric parameter of interest (e.g. weight vs. length/height vs. MUAC) [12]; fieldworker training should therefore cover all measurements in as many anticipated participant types as possible.

This work was conducted in preparation for the multi-site South African National Dietary Intake Survey 2022 (NDIS-2022), which aimed to recruit a nationally representative sample of South African preschoolers, schoolchildren, adults and elderly, with anthropometric data included as indicators of under- and overnutrition in all age groups. Given the aforementioned considerations, evidence that fieldworkers could perform anthropometric measurements with acceptable reliability was considered critical. Following training, such reliability was assumed in previous South African national surveys, but evidence thereof was not documented; post-hoc analysis suggests that anthropometric data quality in these studies was moderate at best [1]. Furthermore, nearly all reports of pre-survey reliability assessment are from high-income country settings, primarily European [3, 13–15] and North American [16, 17], leaving a conspicuous data gap for low-and middle-income countries, particularly sub-Saharan Africa. Additionally, the reliability of calf circumference (CC) measurements in a multi-site survey has not yet been assessed outside of the National Health and Nutrition Examination Surveys that were used to develop the reference standards [18].

### Aim

This study aimed to assess the intra- and inter-rater reliability of site lead anthropometrists and anthropometry fieldworkers for all the anthropometric measurements included in the NDIS-2022 (namely weight, length/ height, MUAC, WC and CC) in volunteers of all the age groups included in the NDIS-2022.

## Methods

Data collection in the NDIS-2022 was decentralised to twelve teams working in nine provinces, each managed by a two-person team (one site lead, who was also responsible for fieldworker training, and one coordinator).

Anthropometry training and reliability assessment were based on a newly developed twelve-module training programme (Table S1). To facilitate international comparability of the NDIS-2022 results, protocols were based on World Health Organisation guidelines for anthropometry in children under five [10], the DHS Programme best-practice guidelines for anthropometric data collection in Demographic and Health Surveys [19], and the FANTA Guide to Anthropometry [20]. The training manual was supplemented by PowerPoint presentations, all of which are available in the public domain [21].

### Training and standardisation of site lead anthropometrists

Orientation and training of the twelve site leads (plus site coordinators) were conducted by the authors (FW, SN and LvdB) during a two-day, centralised meeting (January 2022). This served as a prototype for the decentralised fieldworker training, which the twelve site leads and coordinators would conduct. Most of the site leads and coordinators were dietitians/ nutritionists with prior training in anthropometry. After working through the training manual and accompanying PowerPoint Presentations, the measurement techniques were demonstrated and practised on a variety of adult, child and infant volunteers. The anthropometric equipment purchased for the main study (Table S2) was used.

For logistical, safety and COVID-19-related reasons, the reliability assessment on adult volunteers was conducted at the training venue and on children under five at an Early Child Development Centre. Trainees worked in pairs to take measurements on three infants (aged 7–14 months), three to four children (aged 3–4 years) and three adults. Measurements were documented on a study-specific form, which was submitted after the first round of measurements to reduce recall bias. After completing one round of measurements on all volunteers, the process was repeated on the same volunteers to yield two measurement sets for each trainee pair.

Based on post-training feedback from the site leads, minor adjustments were made to the study protocol (e.g. minimum requirements for volunteer numbers and attributes, and measurement of WC by a fieldworker of the same sex as the participant).

### Training and standardisation of fieldworkers

Site leads and coordinators were responsible for fieldworker training at their respective sites (February 2022), using similar training procedures with minor site-specific adjustments as needed. Eight provincial training sessions were conducted; site trainings were combined where one province contained two study sites (three provinces), or neighbouring provinces had small fieldwork teams (two provinces). Two anthropometry fieldworkers (a lead measurer and an assistant) were appointed to each fieldwork team. In total, 46 pairs of anthropometry fieldworkers were trained (3–11 teams per province, depending on data collection burden; Table S3). Post-training reliability assessment followed procedures similar to the site lead training, with two rounds of measurements completed on the same volunteers. Standardisation volunteers included one or more persons aged 0–1 years, 1–5 years and >12 years (including overweight/ obese adults) to ensure representation of the most challenging measurement conditions. Following preliminary reliability analyses, retraining was required at one site, with markedly lower reliability and an insufficient variety of standardisation volunteers. Only the reliability data collected after the retraining are included in these analyses.

### Data management and analysis

All raw data were captured and cleaned in Excel and analysed using R (R Foundation for Statistical Computing, Vienna, Austria) [22]. Both measurements were used to assess intra-rater reliability, while only the first measurement was used to assess inter-rater reliability. Data from volunteers that were measured by only one fieldworker were excluded, as this does not allow for assessment of inter-rater reliability.

Intra- and inter-rater reliability were assessed using the technical error of measurement (TEM), relative TEM (%TEM), coefficient of reliability (R) and intra-class correlation coefficient (ICC) [12, 23–25] using the equations shown in Table S4. Reliability statistics were calculated separately for each measured parameter (i.e. weight, length/ height, MUAC, WC and CC), for site leads and fieldworkers, and by volunteer age group (0 ≤ 2 years, 2–12 years and >12 years), where relevant.

The TEM indicates the overall absolute error, expressed in the same units as the measurement being analysed (e.g. kg for weight, cm for height) [24]. The difference between the site leads’ and fieldworkers’ TEM was quantified using an F-statistic [*F* = (TEM site leads)^2^ /(TEM fieldworkers)^2^] and *p*-values calculated with N-1 degrees of freedom. Relative TEM describes the TEM as a percentage of the mean of the measurements to compensate for the correlation between TEM and measurement size [24]. Lower TEM and %TEM values indicate greater reliability. The coefficient of reliability (R) quantifies the proportion of observed between-subject variance that is not attributable to measurement error [24]. The ICC incorporates elements of both correlation and agreement between two sets of measurements [25]. The ICC was calculated using R software packages *‘irr’* [26] and *‘irrNA'* [27], using a one-way random-effects model with the measurement from a single rater as the basis of the assessment [25]. Higher ICC and *R* values (up to one) indicate greater reliability.

Bland-Altman analysis was used to visualise agreement using the R software package *‘blandr’* [28]. For intra-rater agreement, the difference between repeated measurements (y-axis) was plotted against the mean of the same two measurements (*x*-axis). For inter-rater agreement, the difference between the first measurement done by the measurer and the mean of all the measurements of the same parameter (*y*-axis) was plotted against the mean of all the measurements of that parameter (*x*-axis). The overall bias (with a 95% confidence interval) was calculated as the mathematical mean of the differences (i.e. *y*-axis values), and the limits of agreement were set at two standard deviations above and below this mean.

### Ethical considerations

Informed consent/ assent was obtained from all participants (and parents/ guardians, where appropriate). The NDIS-2022 received umbrella ethical approval (University of the Western Cape: BM21/4/12). Site-specific ethical approval and institutional permissions were obtained where required.

## Results

The reliability assessment was conducted with 15 volunteers for the site leads and 75 volunteers for the fieldworkers (Table S3). Each volunteer was measured by 2–11 (median 4) different measurers. Intra- and inter-rater TEM, %TEM, R and ICC for all anthropometric parameters are shown in Table 1. According to %TEM, both site leads and fieldworkers had the highest reliability for weight (inter-rater %TEM 0.320–0.923; intra-rater %TEM 0.260–0.645) and length/ height (inter-rater %TEM 0.581–0.855, intra-rater %TEM 0.434–0.757). For fieldworkers, MUAC had the poorest reliability (inter-rater %TEM 3.199; intra-rater %TEM 2.592), while site leads performed poorest at WC (inter-rater %TEM 2.945; intra-rater %TEM 2.353). Analysed by age group, fieldworkers’ %TEM was consistently highest (i.e. least reliable) in the 0 ≤ 2 years group.

**Table 1:** Technical error of measurement (TEM), coefficient of reliability (R) and intraclass correlation coefficient (ICC) for anthropometric measurements: inter-and intra-rater reliability of site lead anthropometrists and fieldworkers.

PARAMETER	MEASURERS AND VOLUNTEERS (*N* = number of volunteers)	INTER-RATER					INTRA-RATER				
		n^a^	TEM^b^	% TEM^c^	R^d^	ICC^e^	n^a^	TEM ^b^	% TEM^c^	R^d^	ICC^e^
Weight (kg)^f^	Fieldworkers, all volunteers (*N* = 75)	321	0.291	0.923	>0.999	>0.999	300	0.215	0.645	>0.999	>0.999
	• 0 ≤ 2 years (*N* = 27)	110	0.198	2.173	0.994	0.995	90	0.182	2.000	0.995	0.995
	• 2–12 years (*N* = 26)	123	0.159	0.934	0.998	0.999	114	0.177	1.022	0.998	0.998
	• >12 years (*N* = 22)	88	0.458	0.603	0.999	0.999	96	0.277	0.368	>0.999	>0.999
	Site leads, all volunteers (*N* = 15)	48	0.097	0.320	>0.999	>0.999	48	0.075	0.260	>0.999	>0.999
	*P*-value^g^: TEM: Site leads vs. fieldworkers	0.012*					<0.001*				
Length/ height (cm)^f^	Fieldworkers, all volunteers (*N* = 74)	315	0.920	0.855	0.999	0.999	296	0.851	0.757	>0.999	0.999
	• 0 ≤ 2 years (*N* = 28)	112	1.165	1.611	0.986	0.987	91	1.146	1.590	0.986	0.986
	• 2–12 years (*N* = 26)	123	0.542	0.522	0.996	0.997	114	0.723	0.695	0.994	0.994
	• >12 years (*N* = 20)	80	0.921	0.569	0.991	0.991	91	0.620	0.381	0.996	0.996
	Site leads, all volunteers (*N* = 15)	48	0.652	0.581	>0.999	>0.999	48	0.478	0.434	>0.999	>0.999
	*P*-value^g^: TEM: Site leads vs. fieldworkers	0.240					0.009*				
Mid-upper arm circumference (cm)^f^	Fieldworkers, all volunteers (*N* = 70)	299	0.675	3.199	0.994	0.992	285	0.556	2.592	0.996	0.996
	• 0 ≤ 2 years (*N* = 26)	106	0.591	3.762	0.881	0.851	91	0.401	2.537	0.948	0.949
	• 2–12 years (*N* = 24)	113	0.582	3.475	0.926	0.920	104	0.376	2.228	0.975	0.975
	• >12 years (*N* = 20)	80	0.859	2.570	0.978	0.976	90	0.809	2.490	0.980	0.980
	Site leads, all volunteers (*N* = 15)	48	0.595	2.845	0.991	0.992	48	0.448	2.179	0.995	0.995
	*P*-value^g^: TEM: Site leads vs. fieldworkers	0.418					0.185				
Waist circumference (cm)^f^	Fieldworkers (*N* = 20)	80	1.745	1.916	0.989	0.987	91	1.344	1.540	0.992	0.990
	Site leads, all volunteers (*N* = 4)	12	2.364	2.945	0.921	0.925	12	1.890	2.353	0.932	0.934
	*P*-value^g^: TEM: Site leads vs. fieldworkers	0.014*					0.777				
Calf circumference (cm)^f^	Fieldworkers (*N* = 19)	73	0.903	2.360	0.921	0.901	84	0.793	2.102	0.944	0.945
	Site leads, all volunteers (*N* = 4)	12	0.618	1.601	0.889	0.896	12	0.363	0.942	0.951	0.952
	*P*-value^g^: TEM: Site leads vs. fieldworkers	0.192					<0.001*				

^a^*n* number of observations.

^b^*TEM* Technical Error of Measurement.

^c^%TEM = relative TEM = (TEM/mean)x100.

^d^*R* = coefficient of reliability = 1 – ([Total TEM]^2^ / SD^2^).

^e^ICC = intraclass correlation coefficient (One way random effects model).

^f^Measurement precision: weight 0.01 kg; rest 0.1 cm.

^g^*F*-test; *F* = (site leads TEM)^2^ / (fieldworkers TEM)^2^; N-1 degrees of freedom.

*p < 0.05: significant difference between TEM of site lead anthropometrists and fieldworkers.

Site leads’ TEM was significantly lower than that of fieldworkers for weight (inter-rater TEM 0.097 vs. 0.291 kg; *p* = 0.012 intra-rater TEM 0.075 vs. 0.215 kg; *p* < 0.001), length/ height intra-rater assessment (0.478 vs. 0.851 cm; *p* = 0.009) and CC intra-rater assessment (0.363 vs. 0.793 cm; *p* < 0.001). For inter-rater assessment of WC, fieldworkers (TEM 1.745 cm) performed significantly better than supervisors (TEM 2.364 cm; *p* = 0.014).

Whole-sample ICC and *R* values exceeded 0.90 for all parameters except site leads’ CC inter-rater reliability (ICC = 0.896, R = 0.889). Upon analysis by age group, fieldworkers’ inter-rater ICC and R for MUAC in children 0 ≤ 2 years was also < 0.9 (ICC = 0.851, *R* = 0.881).

**Fig. 1:**
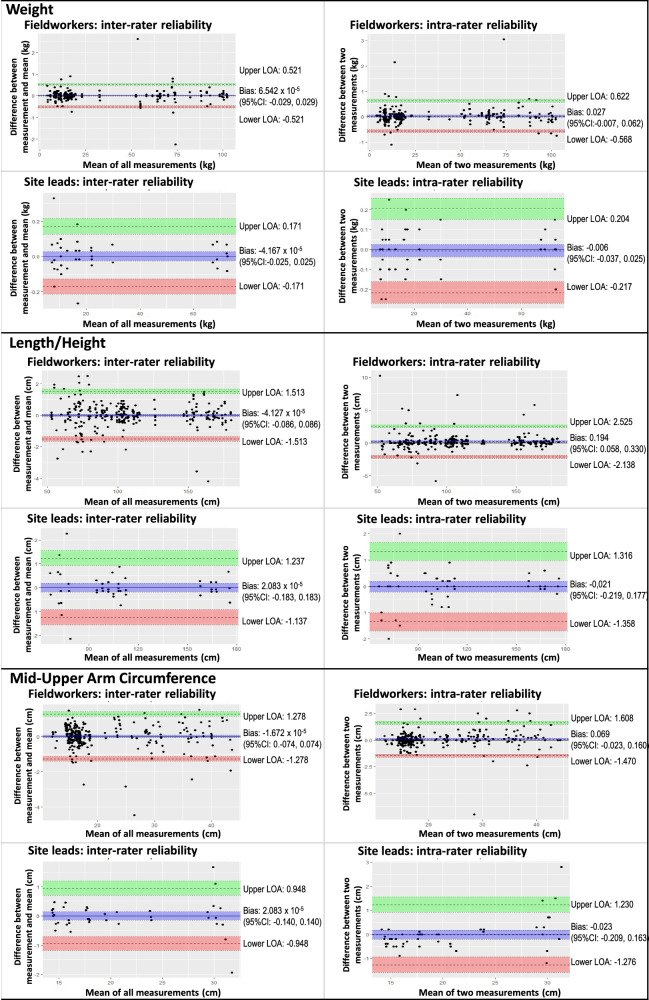
Bland Altman plots illustrating intra- and inter-rater reliability of weight, length/ height and mid-upper arm circumference measurements by fieldworkers and site leads.

**Fig. 2:**
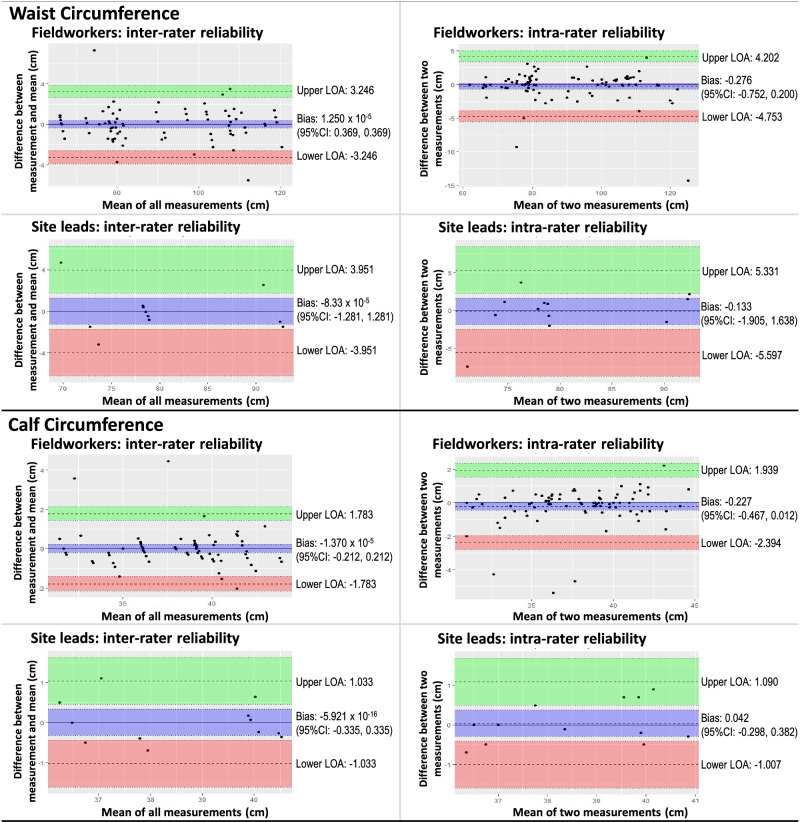
Bland Altman plots illustrating intra- and inter-rater reliability of waist and calf circumference measurements by fieldworkers and site leads.

Bland-Altman plots are shown in Figs. 1 and 2 statistics summarised in Table S5). Statistically significant bias was only seen in fieldworkers’ intra-rater reliability of length/ height measurement (0.194 (0.058, 0.330) cm), attributable to significant bias of 0.220 (0.042, 0.400) cm in the > 12 years group. On all plots, > 90% of the measurements fell within the limits of agreement.

## Discussion

This paper describes the measurement reliability of anthropometry fieldworkers in the South African NDIS-2022, as assessed after standardised training. The NDIS-2022 was the first South African national multi-site nutrition survey to implement this standardised training and reliability assessment approach. Results suggest a high level of reliability among the site lead anthropometrists and lower (though still acceptable) reliability among fieldworkers. Almost all ICC and *R* values exceeded 0.9, indicating excellent reliability [25]. However, %TEM indicated lower reliability for MUAC, CC AND WC than for weight and length/ height.

Few guidelines for acceptable TEM values exist. Absolute TEM is proportionate to the size of the measured value; thus, acceptability thresholds might vary by age group (e.g. infants vs. adults) and measurement (e.g. MUAC vs. length/ height). Using %TEM mitigates this by expressing TEM relative to the size of the measurement, which may allow for consistent cutoffs across various measurements. Carlsey et al. [16] used %TEM < 2.0 for weight and length measurements in children 0–18 years, which our study achieved in all cases except fieldworkers’ weight measurements in children 0 ≤ 2 years. Though not included in the Carlsey et al. study, %TEM for MUAC was > 2 in all groups in our study, suggesting higher measurement variability. In adults, Perini et al. [23] proposed stricter %TEM cutoffs for weight and height, with different standards for beginner (inter-rater %TEM < 2, intra-rater %TEM < 1.5) and experienced (inter-rater %TEM < 1.5, intra-rater %TEM < 1.0) anthropometrists. By this standard, both site leads and fieldworkers had excellent reliability for adult weight and height measurements. Perini et al. did not include MUAC, CC or WC in their guidelines, but the higher %TEM for these measurements suggest greater variability than for weight and height.

Previous studies from primarily high-income countries (Table 2) have described inter- and intra-rater reliability of anthropometric measurements in children [13, 15–17], adolescents [14, 16], adults [15, 29] and the elderly [3]. Whilst exact results vary, some patterns are evident. Firstly, intra-rater reliability consistently exceeded inter-rater reliability, a pattern that held true in this study. Secondly, weight measurements consistently had the highest reliability, and WC (when measured) the lowest [3, 13, 15, 17]. In this study, the reliability of weight and length/height measurements exceeded that of MUAC, CC and WC. This is consistent with the technical difficulty of circumference measurements as well as the novelty of the techniques – particularly CC, which was novel to all the site leads and fieldworkers.

**Table 2:** Summary of reliability statistics in international published research.

Study	Setting	Age range	Measurement	Inter-rater			Intra-rater		
				TEM	%TEM	R or ICC	TEM	%TEM	R or ICC
De Onis [12]	Brazil (WHO Multicentre Growth Reference Study)	0–5 years	Length/ height	2.5	–	–	–	–	–
			MUAC	1.8	–	–	–	–	–
Li [17]	Alaska, Hawaii, Micronesia (Children’s Healthy Living Programme)	2–8 years	Weight	1.69	–	*R* = 0.938	0.03	–	–
			Height	1.44	–	*R* = 0.984	0.28	–	–
			Waist Circumference^a^	2.02	–	*R* = 0.936	0.32	–	–
De Miguel-Etayo [13]	Germany, Netherlands (ToyBox Study)	4–6 years	Weight	0.07	0.40	*R* = 0.9990	0.02	0.10	*R* = 0.9998
			Height	0.82	0.71	*R* = 0.9809	0.12	0.11	*R* = 0.9993
			Waist Circumference^c^	0.81	1.52	*R* = 0.9830	0.49; 0.68	1.08; 1.29	*R* = 0.9453; 0.9673
Carsley [16]	Canada	0–18 years	Weight 0 ≤ 2 yr	0.06	0.64	*R* = 0.9992	0.05	0.61	*R* = 0.9992
			Weight 2 ≤ 5 yr	0.10	0.65	*R* = 0.9985	0.10	0.62	*R* = 0.9987
			Weight 5 ≤ 18 yr	0.17	0.70	*R* = 0.9991	0.17	0.70	*R* = 0.9991
			Length 0 ≤ 2 yr	0.73	1.03	*R* = 0.9906	0.46	0.64	*R* = 0.9963
			Height 2 ≤ 5 yr	0.69	0.70	*R* = 0.9933	0.27	0.27	*R* = 0.9990
			Height 5 ≤ 18 yr	0.45	0.36	*R* = 0.9983	0.24	0.19	*R* = 0.9995
Nagy [14]	Spain, Hungary (HELENA study)	Adolescents	MUAC	1.01	–	*R* = 0.9009	0.13–0.46	–	*R* = 0.9903–0.9990
			Waist Circumference^c^	1.69	–	*R* = 0.9052	0.37–1.75	–	*R* = 0.9793–0.9997
Androustos [15]	Belgium (Feel4Diabetes study)	Primary school children	Weight	0.06	–	*R* = 0.9996	0.14	–	*R* = 0.9976
			Height	0.27	–	*R* = 0.9978	0.27	–	*R* = 0.9977
		Adults	Weight	1.49	–	*R* = 0.9841	0.18	–	*R* = 0.9998
			Height	0.29	–	*R* = 0.9982	0.27	–	*R* = 0.9983
			Waist Circumference^b^	2.50	–	*R* = 0.9556	0.81	–	*R* = 0.9953
Geeta [29]	Malaysia (National Health and Morbidity Survey 2006)	Adults	Weight	0.45	0.75	ICC = 0.999	0.31	0.53	ICC = 0.999
			Height	0.32	0.20	ICC = 0.996	0.32	0.20	ICC = 0.999
			Waist Circumference^b^	0.77	0.91	ICC = 0.999	0.44	0.52	ICC = 0.999
Gomez-Cabello [3]	Spain (EXERNET study)	Elderly ≥ 65 years	Height	0.61		*R* = 0.9946	0.02–0.16	0.02–0.13	*R* = 0.9991–0.9999
			Waist Circumference^d^	3.09		*R* = 0.9637	0.16–1.01	0.17–1.05	*R* = 0.9912–0.9999

Superscript numbers refer to the reference list.

^a^Waist circumference measured at the level of the umbilicus.

^b^Waist circumference measured at the midpoint between the lowest point of the last rib and highest point of the iliac crest.

^c^Waist circumference measurement site not specified.

^d^Waist circumference measured at the narrowest waist between the lowest point of the last rib and highest point of the iliac crest.

Site leads displayed better reliability than fieldworkers for most measurements, although TEM was only significantly different for weight (intra- and inter-rater reliability), length/height (intra-rater reliability) and CC (intra-rater reliability). This may be because most of the site leads (unlike fieldworkers) were dietitians/ nutritionists with prior tertiary-level training in anthropometric assessment. Site leads’ psychological investment and sense of ownership as project leaders may also have inspired more meticulous measurement practices. Unexpectedly, the fieldworkers outperformed the site leads for WC measurements, although only inter-rater TEM differed significantly. This underscores the importance of training and standardisation even if anthropometry is done by qualified persons as, for example, institutions differ in terms of WC site identification protocols, resulting in different values [4].

Bland-Altman analyses revealed no statistically significant bias, except in fieldworkers’ intra-rater reliability of height measurement in subjects > 12 years old, though the magnitude of the bias was small (0.22 (0.042–0.400) cm). Visual inspection of Bland-Altman plots shows greater variability of length/ height measurements in subjects < 100 cm tall (i.e. infants and young children), which is consistent with the technical difficulty of measuring length/ height in this age group. No other age-related trends were evident. In general, acceptable group-level measurement agreement was achieved within and between raters for all the pertinent anthropometric parameters, as is relevant for a national study.

The data described here provides statistical evidence of the ability of NDIS-2022 site leads and fieldworkers to perform anthropometric measurements reliably. The use of consistent training materials, standardised measurement protocols, and identical brand-new equipment across all study sites further contributes to anthropometric data quality. Additionally, this study reports the first South African reliability data for adult CC measurement. This study, alongside the publicly available training materials, paves the way for consistent quality standards for anthropometry in future large-scale South African studies, thus contributing to harmonisation and comparability of data over time and across different settings.

Some limitations must be acknowledged. First, we did not assess measurement accuracy (i.e. how closely the measured values approximate the true value). Obtaining true “gold standard” anthropometric measurements requires highly trained and accredited anthropometrists, which were unavailable in this setting. Careful equipment selection with daily verification checks should minimise equipment-related errors, but systematic errors due to suboptimal measurement technique cannot be ruled out. Finally, limited numbers of trainees and volunteers in some provinces and age groups increase statistical volatility. The pooling of data from all sites allowed for meaningful statistical analyses but may have obscured some inter-site differences.

In line with international recommendations [1, 10, 19], standardised anthropometric training and reliability assessment should precede any large nutrition survey. This increases confidence in the study results (or, conversely, highlights limitations to consider when interpreting the data). Our experience suggests that two days of training are likely insufficient for fieldworkers with no previous anthropometry experience, and more time should be allotted, particularly for hands-on practice. Adding a pre-training assessment of measurement skills would provide evidence of training effectiveness and allow for refinement of the training approach based on trainees’ strengths and challenge areas. During data collection, ongoing reliability assessment must be incorporated to ensure that data quality is maintained. Finally, statistical evidence of anthropometric data quality should be reported in detail alongside the main study results. For child anthropometry, the guidelines set out by the WHO [10] should be followed, and calculation of the composite index of anthropometric data quality described by Perumal et al. [1] is recommended. Communication of data quality (or the absence thereof), as well as steps taken to identify and manage errors, are prerequisites for ethical and transparent science communication and essential for continued trust in science [30]. This empowers both researchers (by allowing re-analyses of existing data, knowledge synthesis, and study reproduction) and policymakers (by allowing meaningful longitudinal monitoring of population trends) [30].

## Conclusion

Using a variety of statistical techniques, this study describes the intra- and inter-rater reliability of anthropometric measurements in the NDIS-2022. It is the first published evidence of its kind for a large-scale multicentre South African survey. Reliable measurement is the basis of anthropometric data quality in nutrition surveys and a prerequisite for valid results. While most of the raw anthropometric data showed acceptable reliability, consistent measurement precision cannot be assumed even for well-established measurements (e.g. infant length, MUAC and WC). This highlights the importance of harmonisation of measurement protocols, intensive pre-survey training and objective and continuous assessment of reliability.

## Supplementary information

Table S1

Table S2

Table S3

Table S4

Table S5

## Data Availability

Data may be obtained from the authors upon reasonable request.
