# Supplementary material for: Statistical assessment of reliability of anthropometric measurements in the multi-site South African National Dietary Intake Survey 2022
Source: Eur J Clin Nutr. 2024 May 14;78(11):1005–13. doi: 10.1038/s41430-024-01449-1 (PMC11537951; doi:10.1038/s41430-024-01449-1)
Supplement: Supplementary file 1 — Table S1 [file 41430_2024_1449_MOESM1_ESM.docx]

Table S1: Overview of training modules

| Module 1: | Introduction to anthropometry, including a brief introduction to the equipment ^a^ |
| --- | --- |
| Module 2: | Anthropometric equipment: calibration, verification, care and maintenance |
| Module 3: | Anthropometry-specific infection prevention and control ^b^ |
| Module 4: | Pre-measurement procedures |
| Module 5: | General guidelines for measuring and recording |
| Module 6: | Measuring weight |
| Module 7: | Measuring length/ height |
| Module 8: | Measuring mid-upper arm circumference |
| Module 9: | Measuring calf circumference |
| Module 10: | Measuring waist circumference |
| Module 11: | Conducting the training ^c^ |
| Module 12: | Conducting the standardisation and reliability assessment ^c^ |
| The full training manual and PowerPoint presentations are available on <https://www.up.ac.za/centre-for-maternal-fetal-newborn-and-child-healthcare/article/3043272/anthropometry-body-composition-and-growth-assessment> . | |
| ^a^ Module 1 was presented online for capacity development all fieldworkers to view on demand, including fieldworkers not designated to perform anthropometric measurements  ^b^ Module 3 included measures related to the prevention of transmission of COVID-19  ^c^ Modules 11 and 12 were only presented during the training of the site lead anthropometrists, not during fieldworker training | |
