# Supplementary material for: Statistical assessment of reliability of anthropometric measurements in the multi-site South African National Dietary Intake Survey 2022
Source: Eur J Clin Nutr. 2024 May 14;78(11):1005–13. doi: 10.1038/s41430-024-01449-1 (PMC11537951; doi:10.1038/s41430-024-01449-1)
Supplement: Supplementary file 2 — Table S2 [file 41430_2024_1449_MOESM2_ESM.docx]

Table S2: Anthropometric equipment used in the South African National Dietary Intake Survey

| Anthropometric parameter | Equipment details | | | |
| --- | --- | --- | --- | --- |
|  | Brand | Range | Gradation | Precision |
| Weight  (≤2 years: tared weight) | Seca ^a^ 874 electronic platform scales | 0-200 kg | 0-150 kg: 50g  150-200 kg: 100 g | 0-50 kg: ±100 g  50-200 kg: ±0.15% |
| Infant length (≤2 years) | Seca ^a^ 417 infantometer | 10-100 cm | 1 mm | ±5 mm |
| Child and adult height | Seca ^a^ 231 stadiometer | 20-205 cm | 1 mm | ±5 mm |
| MUAC^b^ : <5 years | ShorrTape^c^ colour coded MUAC insertion tape | 6-26 cm | 1 mm | Not specified |
| MUAC^b^ : ≥5 years | Seca ^a^ 201 spring-wound tape measure | 205 cm | 1 mm | ±5 mm |
| Waist circumference |  |  |  |  |
| Calf circumference |  |  |  |  |
| ^a^ Seca measurement systems, Hamburg, Germany  ^b^ MUAC: mid-upper arm circumference  ^c^ Weigh and measure LLC, Olney, MD, USA | | | | |
