# Supplementary material for: Statistical assessment of reliability of anthropometric measurements in the multi-site South African National Dietary Intake Survey 2022
Source: Eur J Clin Nutr. 2024 May 14;78(11):1005–13. doi: 10.1038/s41430-024-01449-1 (PMC11537951; doi:10.1038/s41430-024-01449-1)
Supplement: Supplementary file 3 — Table S3 [file 41430_2024_1449_MOESM3_ESM.docx]

Table S3: Number of teams trained, and number of volunteers used for standardisation, per province.

| Team code | Number of teams^a^ trained | Number of volunteers | | |
| --- | --- | --- | --- | --- |
|  |  | 0-2 years | 2-12 years | >12 years |
| Province A | 3 | 2 | 2 | 5 |
| Province B | 3 | 3 | 2 | 3 |
| Province C | 11 | 7 | 3 | 1 ^b,c^ |
| Province D | 6 | 1 | 5 | 2 |
| Province E | 7 | 2 | 3 | 2 |
| Province F | 4 | 2 | 1 | 3 |
| Province G | 4 | 2 | 2 | 4 |
| Province H | 8 | 6 | 8 | 4 |
| Total | **46** | **25** | **26** | **24** |
| Site lead anthropometrists | 6 | 4 | 7 | 4 |
| ^a^ Each team consisted of two people  ^b^ 6 additional volunteers (not included in this total) only attended one round of measurements; their data was used for assessment of inter-rater reliability only.  ^c^ Measurements for 10 additional volunteers were excluded from the analysis as they were each only measured by one fieldworker | | | | |
