# Supplementary material for: Statistical assessment of reliability of anthropometric measurements in the multi-site South African National Dietary Intake Survey 2022
Source: Eur J Clin Nutr. 2024 May 14;78(11):1005–13. doi: 10.1038/s41430-024-01449-1 (PMC11537951; doi:10.1038/s41430-024-01449-1)
Supplement: Supplementary file 4 — Table S4 [file 41430_2024_1449_MOESM4_ESM.docx]

Table S4: Calculation of reliability statistics

| Statistic | Formula | Variables |
| --- | --- | --- |
| Technical Error of Measurement (TEM) | $TEM= \sqrt{\frac{\sum_{1}^{N} \left( \sum_{1}^{K} M^{2}- \frac{({\sum_{1}^{K} M)}^{2}}{K} \right)}{N\left( K-1 \right)}}$ | *N* = number of volunteers,  *K* = number of measurers,  *M* = individual measured values |
| Relative TEM (%TEM) | $\% TEM= {(TEM}/{mean)*100}$ | *mean* = mathematical mean of all individual measured values |
| Coefficient of reliability (R) | $R=1-\left( {{TEM}^{2}}/{{SD}^{2}} \right)$ | *SD^2^* = overall variance of all individual measured values |
