# Supplementary material for: Statistical assessment of reliability of anthropometric measurements in the multi-site South African National Dietary Intake Survey 2022
Source: Eur J Clin Nutr. 2024 May 14;78(11):1005–13. doi: 10.1038/s41430-024-01449-1 (PMC11537951; doi:10.1038/s41430-024-01449-1)
Supplement: Supplementary file 5 — Table S5 [file 41430_2024_1449_MOESM5_ESM.docx]

Table S5: Bland-Altman statistics for anthropometric measurements: inter- and intra-rater reliability of site lead anthropometrists and fieldworkers

| PARAMETER | MEASURERS AND VOLUNTEERS  (N=number of volunteers) | INTER-RATER RELIABILITY | | | | | INTRA-RATER RELIABILITY | | | | |
| --- | --- | --- | --- | --- | --- | --- | --- | --- | --- | --- | --- |
|  |  | n ^a^ | Bias ^b^ (95% CI) | Limits of agreement ^c^ | Outliers ^d^  n (%) | | n ^a^ | Bias ^b^ (95% CI) | Limits of agreement ^c^ | Outliers ^d^  n (%) | |
| Weight  (kg) | Fieldworkers, all (N=75) | 321 | 6.5x10^‑5^ (-0.029, 0.029) | -0.521; 0.521 | 9 | (2.8) | 300 | 0.027 (-0.007, 0.062) | -0.568; 0.622 | 13 | (4.3) |
|  | - 0-<2 years (N=27) | 110 | 5.5x10^‑5^ (-0.031, 0.031) | -0.322; 0.322 | 7 | (6.4) | 90 | 0.006 (-0.048, 0.060) | -0.501; 0.512 | 5 | (5.6) |
|  | - 2-12 years (N=26) | 123 | 4.9x10^‑5^ (-0.025, 0.025) | -0.276; 0.276 | 3 | (2.4) | 114 | 0.032 (-0.014, 0.078) | -0.456; 0.521 | 3 | (2.6) |
|  | - >12 years (N=22) | 88 | 1.0x10^‑4^ (-0.094, 0.095) | -0.873; 0.874 | 2 | (2.3) | 96 | 0.042 (-0.037, 0.121) | -0.725; 0.808 | 1 | (1.0) |
|  | Site leads , all (N=15) | 48 | -4.2x10^‑5^ (-0.025, 0.025) | -0.171; 0.171 | 3 | (6.3) | 48 | -0.006 (-0.037, 0.025) | -0.217; 0.204 | 3 | (6.3) |
| Length/ height  (cm) | Fieldworkers, all (N=74) | 315 | ‑4.1x10^‑5^ (-0.086, 0.086) | -1.513; 1.513 | 21 | (6.7) | 296 | 0.194 (0.058, 0.330)* | -2.138; 2.525 | 12 | (4.1) |
|  | - 0-<2 years (N=28) | 112 | ‑1.8x10^‑5^ (-0.186, 0.186) | -1.943; 1.943 | 8 | (7.1) | 91 | 0.237 (-0.098, 0.573) | -2.924; 3.398 | 4 | (4.4) |
|  | - 2-12 years (N=26) | 123 | -4.1x10^‑5^ (-0.084, 0.084) | -0.927; 0.927 | 6 | (4.9) | 114 | 0.138 (-0.051, 0.326) | -1.856; 2.132 | 3 | (2.6) |
|  | - >12 years (N=20) | 80 | -7.5x10^‑5^  (-0.179, 0.178) | -1.572; 1.572 | 2 | (2.5) | 91 | 0.220 (0.042, 0.398) * | -1.454; 1.894 | 2 | (2.2) |
|  | Site leads, all (N=15) | 48 | 2.1x10^‑5^ (-0.183, 0.183) | -1.237; 1.237 | 3 | (6.3) | 48 | -0.021 (-0.219, 0.177) | -1.358; 1.316 | 3 | (6.3) |
| Mid-upper arm circumference  (cm) | Fieldworkers, all (N=70) | 299 | -1.7x10^‑5^ (-0.074, 0.074) | -1.278; 1.278 | 14 | (4.7) | 285 | 0.069 (-0.023, 0.160) | -1.470; 1.608 | 15 | (5.3) |
|  | - 0-<2 years (N=26) | 106 | -4.7x10^‑5^ (-0.112, 0.112) | -1.136; 1.136 | 3 | (2.8) | 91 | 0.012 (-0.107, 0.131) | -1.105; 1.129 | 6 | (6.6) |
|  | - 2-12 years (N=24) | 113 | -1.6x10^‑17^ (-0.113, 0.113) | -1.193; 1.193 | 3 | (2.7) | 104 | 0.023 (-0.081, 0.127) | -1.024; 1.070 | 5 | (4.8) |
|  | - >12 years (N=20) | 80 | -4.4x10^‑17^ (-0.177, 0.177) | 1.558; 1.558 | 3 | (3.8) | 90 | 0.179 (-0.059, 0.417) | -2.048; 2.406 | 5 | (5.6) |
|  | Site leads, all (N=15) | 48 | 2.1x10^‑5^ (-0.140, 0.140) | -0.948; 0.948 | 3 | (6.3) | 48 | -0.023 (-0.209, 0.163) | -1.276; 1.230 | 3 | (6.3) |
| Calf circumference  (cm) | Fieldworkers (N=19) | 73 | -1.4x10^-5^ (-0.212, 0.212) | -1.783; 1.783 | 3 | (4.1) | 84 | -0.227 (-0.467, 0.012) | -2.394; 1.939 | 4 | (4.8) |
|  | Site leads (N=4) | 12 | -5.9x10^‑16^ (-0.335, 0.335) | -1.033; 1.033 | 1 | (8.3) | 12 | 0.042 (-0.298, 0.382) | -1.007; 1.090 | 0 | (0.0) |
| Waist circumference  (cm) | Fieldworkers (N=19 | 80 | 1.3x10^‑5^ (-0.369, 0.369) | -3.246; 3.246 | 4 | (5.0) | 91 | -0.276 (-0.752, 0.200) | -4.753; 4.202 | 3 | (3.3) |
|  | Site leads (N=4) | 12 | -8.3x10^‑5^ (-1.281, 1.281) | -3.951; 3.951 | 1 | (8.3) | 12 | -0.133 (-1.905, 1.638) | -5.597; 5.331 | 1 | (8.3) |
| ^a^ n = number of observations  ^b^ Bias: the mean of the differences between round one and round two measurements  ^c^ Limits of agreement: two standard deviations (of the differences between round one and round two measurements) above and below the bias  ^d^ Outliers: number (%) of values that fall outside the limits of agreement  * Significant bias: the 95% CI of the bias does not include 0 | | | | | | | | | | | |
